# A novel immune-related genes signature after bariatric surgery is histologically associated with non-alcoholic fatty liver disease

**DOI:** 10.1080/21623945.2021.1970341

**Published:** 2021-09-10

**Authors:** Yancheng Song, Jan Zhang, Hexiang Wang, Dong Guo, Chentong Yuan, Bo Liu, Hao Zhong, Dongmei Li, Yu Li

**Affiliations:** a Department of Gastrointestinal Surgery, The Affiliated Hospital of Qingdao University, Qingdao, China; b Department of Colonretal Surgery, The Affiliated Hospital of Qingdao University, Qingdao, P.R. China; c Department of Radiology, The Affiliated Hospital of Qingdao University, Qingdao, China

**Keywords:** Bariatric surgery, nafld, immune-related gene, adipose tissue, obesity

## Abstract

Increasing evidence shows that immune-related genes (IRGs) play an important role in bariatric surgery (BS). We identified differentially expressed immune-related genes (DEIRGs) of adipose tissue after BS by analysing the two expression profiles of GEO (GSE59034 and GSE29409). Subsequently, enrichment analysis, GSEA and PPI networks were examined to identify the hub IRGs and related pathways. The performance of the signature was evaluated by area under the curve (AUC) of the receiver operating characteristic (ROC). CIBERSORT algorithm was used to evaluate the relative abundance of infiltrated immune cells.42 DEIRGs were found between the GSE59034 and GSE29409 datasets. The AUC of the signature was 0.904 and 0.865 in the GSE58979 and GSE48452, respectively. Interestingly, the signature also showed good performance in diagnosing non-alcoholic fatty liver disease (NAFLD) (AUC was 0.834 and 0.800, respectively). The number of neutrophils, macrophages M2, macrophages M0 and dendritic cells activated decreased significantly. After BS, the infiltration of T cells regulatory, monocytes, mast cells resting and plasma cells in adipose tissue increased. The novel proposed IRGs signature reveals the underlying immune mechanism of BS and is a promising biomarker for distinguishing the severity of NAFLD. This will provide new insights into strategies for treating obesity and NAFLD.

## Introduction

Globally, from 1975 to 2016, the number of obese patients (age >5 years) increased from 111 million to 797 million [1]. Obesity has clearly become a global burden with serious public health implications, and obesity-related metabolic diseases are spreading rapidly as obesity increases [2,3]. Obesity is closely related to non-alcoholic fatty liver disease (NAFLD) [4]. NAFLD is a clinicopathological stage from hepatic steatosis to non-alcoholic steatohepatitis (NASH) and cirrhosis [5]. With the rise of obesity, the prevalence of NAFLD has risen sharply [6]. Currently, the global prevalence of NAFLD can reach 24% [7]. Adipose tissue is an endocrine organ involved in the regulation of energy and inflammation [8]. Study has shown that adipose tissue inflammation is a prerequisite for the development of NAFLD in mice [9]. The immune cells infiltrating into fat can regulate the production and secretion of pro-inflammatory and anti-inflammatory factors in adipose tissue to induce NAFLD [9,10].

Currently, bariatric surgery (BS) is a more effective treatment for morbid obesity and NAFLD than non-surgical treatment [11]. Some studies have also reported changes in the metabolic and immune status of adipose tissue following BS [10,12]. BS can promote the significant transformation of adipose tissue from pro-inflammatory state to anti-inflammatory state, and ameliorate adipose tissue function [13]. In addition, BS results in sustained weight loss and may reduce liver fat, inflammation, and fibrosis, reversing changes in hepatic pathology in patients with NAFLD and NASH [14]. However, the relationship between adipose tissue and NAFLD after BS remains unclear. It can be concluded from previous studies that immune-related genes (IRGs) play an important role in BS. Therefore, the analysis of IRGs in adipose tissue after BS is of great significance for exploring the potential value of new biomarkers for NAFLD.

With the rapid development of gene sequencing technology, IRG-based signatures have been constructed and validated in different types of diseases [15,16]. Some of these IRGs signatures have good sensitivity and specificity in predicting patient prognosis and can be used as potential tools to guide individualized therapy. However, IRGs signatures for clinical application have not been developed for BS.

In this study, we investigated the potential immune mechanism of BS in ameliorating NAFLD through a comprehensive bioinformatics analysis of adipose tissue. A novel IRGs signature was constructed and used to predict the histological degree of NAFLD. This study provides new insights into the immune regulation mechanism of BS and provides information for personalized diagnosis and treatment of NAFLD.

## Methods

### Microarray data

The gene expression profile was obtained from the GEO database (http://www.ncbi.nlm.nih.gov/geo). Gene expression profiles GSE59034 [17], GSE29409 [18], GSE83452 [19], GSE58979 [20] and GSE48452 [21] were selected from the GEO database (Table 1). The platform of GSE59034 was GPL11532 [HuGene-1_1-st] Affymetrix Human Gene 1.1 ST Array (Affymetrix, Inc., Santa Clara, CA). A total of 32 adipose tissue samples were obtained from patients before BS (n = 16) and patients after BS (n = 16) for subsequent analysis. The platform of GSE29409 was GPL7020, NuGO array (human) NuGO_Hs1a520180. Adipose tissues were obtained from five before BS patients and five after BS patients, with a total of 10 samples for subsequent analysis. The platform of GSE83452 was GPL16686 [hugene-2_0-ST] Affymetrix Human Gene 2.0 ST Array [transcript (Gene) version]. A total of 76 liver tissue samples were obtained from GSE83452. The platform of GSE58979 was GPL15207 [PrimeView] Affymetrix Human Gene Expression Array. A total of 53 adipose tissue specimens were obtained. According to histology, the samples were divided into group I (<5% steatosis), group II (NAFLD, 30–50% steatosis), group III (NASH) and group IV (NASH + fibrosis). The platform of GSE48452 was GPL11532 [HuGene-1_1-st] Affymetrix Human Gene 1.1 ST Array [transcript (gene) version]. A total of 73 liver tissue samples were obtained from GSE48452. Series matrix files and platform files for GSE59034, GSE29409, GSE83452, GSE58979 and GSE48452 were downloaded. All the data are from the open access GEO database, so we do not need Ethics Committee approval for our study.

**Table 1.** Characteristics of the included microarray datasets

GSE ID	Participants	Tissues	Year	Analysis type	Platform
GSE59034	16 before BS and 16 after BS	Adipose tissue	2017	Array	GPL11532
GSE29409	5 before BS and 5 after BS	Adipose tissue	2012	Array	GPL7020
GSE83452	76 samples	Liver tissue	2017	Array	GPL16686
GSE58979	53 samples	Adipose tissue	2015	Array	GPL15207
GSE48452	73 samples	Liver tissue	2013	Array	GPL11532

### Identification of differentially expressed genes (DEGs)

GEO2R (http://www.ncbi.nlm.nih.gov/geo/geo2r/) is the tool used to identify DEGs in the GEO database. GEO2R was used to identify DEGs before and after BS. |log FC| >0.5 and p < 0.05 were used as the threshold for DEGs screening.

### Differentially expressed immune-related genes (DEIRGs)

IRGs were downloaded from the ImmPort database (https://immport.niaid.nih.gov). The overlapping DEIRGs were selected from DEGs and IRGs for further analysis. The online tool Venny (https://bioinfogp.cnb.csic.es/tools/venny/index.html) was used to select the common DEIRGs of the two datasets.

### Functional and pathway enrichment analysis

DAVID database (DAVID, http://david.abcc.ncifcrf.gov/) is considered to be the most commonly used function annotation tool [22]. David was used for biofunctional analysis, including Gene Ontology (GO) analysis and Kyoto Encyclopaedia of Genes and Genomes (KEGG) pathway enrichment analysis. DEIRGs were uploaded to investigate their potential functions. The cut-off criteria for p-value and false discovery rate (FDR) were 0.05 for both KEGG and GO analysis.

### Gene Set Enrichment analysis (GSEA)

Further GSEA were performed on all IRGs to detect significant differences in biological functions resulting from BS. The R package ‘fgsea’ was used for GSEA, and 0.05 was the cut-off P value of the GSEA [23]. Ten thousand times per analysis was performed for gene set.

### Protein–Protein Interaction (PPI) network analysis and identification of hub IRGs

Hub genes are generally considered to be functionally critical among other genes. We uploaded the DEIRGs to the STRING database (http://www.string-db.org/) and then selected the confidence >0.4 as the cut-off criterion for PPI network analysis. Cytoscape software was used to plot PPI network. Cytohubba plug-ins in Cytoscape was used to study key nodes in the PPI network [24]. The degree method was used to identify the hub genes in PPI network. The top 10 genes were identified as hub IRGs.

### IRGs signature construction and validation

Hub IRGs between preoperative and postoperative BS were identified from GSE59034 and GSE29409. Logistic regression was applied to establish IRGs signature for predicting the histological severity of NAFLD, and all hub IRGs were covariates. GSE58979 (n = 53) and GSE48452 (n = 32) were used for signature validation. According to the histological severity of NAFLD, 53 patients from GSE58979 were divided into mild steatosis group (group I and group II) and severe steatosis group (group III and group IV). The performance of the signature was evaluated by area under the curve (AUC) of the receiver operating characteristic (ROC) and consistency index (C index). The Hosmer–Lemeshow test was used to evaluate goodness-of-fit of the signature. Decision curve analysis (DCA) was used to quantify the net benefit of patients under different threshold probabilities to determine the clinical value of signature.

### Construction of a diagnostic signature for NAFLD

To investigate whether IRGs signature could be used to predict the occurrence of NAFLD, GSE48452 (n = 73) and GSE83452 (n = 76) were used to validate the signature. The predictability of the signature was then evaluated by AUC of ROC and DCA. Goodness-of-fit was examined by using the Hosmer–Lemeshow test.

### Comparison of immune cells infiltration in preoperative and postoperative BS

CIBERSORT algorithm was used to evaluate the relative abundance of infiltrated immune cells before and after BS. CIBERSORT can estimate the composition of 22 immune cell types, mainly including plasma cells, macrophages, eosinophils, natural killer cells, B cells, dendritic cells, T cells, and neutrophils [25]. The R package ‘CIBERSORT’ was used. Wilcox test was used to compare the differences of immune cell types between the pre-BS and the post-BS.

## Results

### DEIRGs after bariatric surgery

**Figure 1.**
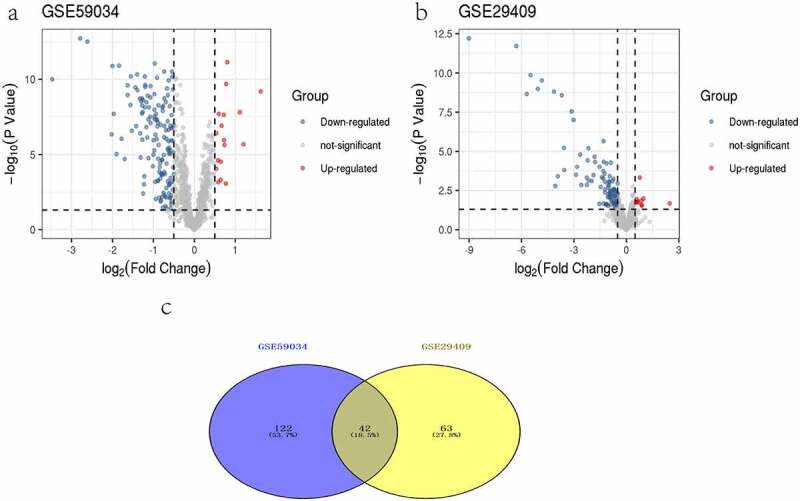
Volcano plots of differentially expressed genes. (a) GSE59034, (b) GSE29409. Data points in red represent up-regulated, and blue represent down-regulated genes. The differences are set as |log FC|>0.5. (c)Venn diagram of common differentially expressed genes from the two datasets

1162 DEGs were identified from the GSE59034 dataset, including 272 upregulated DEGs and 890 downregulated DEGs with |log FC| >0.5 and p < 0.05. Six hundred and fifty-eight DEGs were identified from the GSE29409 dataset, including 184 upregulated DEGs and 474 downregulated DEGs with |log FC| >0.5 and p < 0.05. IRGs were extracted from the ImmPort database. Next, DEIRGs were determined between the extracted IRGs and DEGs. A total of 164 DEIRGs were identified from the GSE59034 dataset (Figure 1a), which 17 were upregulated and 147 were downregulated. A total of 105 DEIRGs were identified from the GSE29409 dataset (Figure 1b), which 10 were upregulated and 95 were downregulated. We used the online tool Venny to select 42 co-expressed DEIRGs (Figure 1c and Table 2).

**Table 2.** 42 DEIRGs after bariatric surgery

Regulation	Gene Symbol
Down	AQP9, C3AR1, C5AR1, CCL18, CCL2, CCR1, CD48, CSF2RB, CSF3R, CTSS, CXCL2, CXCR4, EDNRB, FCER1G, FGF1, FGR, FPR1, HCK, HMOX1, IL10, IL10RA, IL12 RB2, IL6, LYN, NR4A3, PIK3CG, PLXNC1, PRKCB, PTAFR, PTPN6, RAC2, S100A12, S100A8, STC1, TFRC, THBS1, TLR1, TLR2, TLR8, TNFAIP3, TNFSF13B, VAV1

### Biofunctional analysis of the DEIRGs

**Figure 2.**
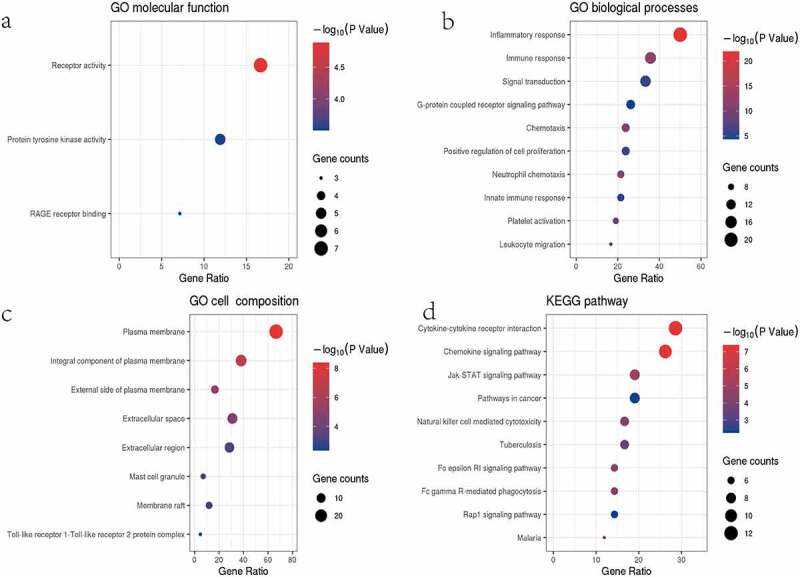
Results of the GO (a–c) and KEGG (d) analyses. (a-c) The x-axis label represents the gene ratio, and the y-axis label represents GO terms. (d) The x-axis label represents the gene ratio, and the y-axis label represents pathway. Different colour of circle represents different p value. The size of circle (gene counts) represents amount of DEGs enriched in pathway. Gene ratio represents amount of DEGs enriched in the pathway/amount of all DEGs in background gene set

The biological functions of 42 DEIRGs were studied by GO and KEGG analysis (Figure 2). In GO analysis, for molecular function (MF), these genes were significantly enriched in receptor activity, protein tyrosine kinase activity, and RAGE receptor binding. For biological process (BP), these genes were significantly enriched in signal transduction, G-protein coupled receptor signalling pathway, chemotaxis, positive regulation of cell proliferation and neutrophil chemotaxis. And they played a key role in host inflammatory response, immune response, and innate immune response. For cellular component (CC), these genes were enriched in plasma membrane, integral component of membrane, external side of plasma membrane, extracellular space, extracellular region, mast cell granule, membrane raft, and Toll-like receptor 1-Toll-like receptor 2 protein complex. KEGG analysis (Figure 2d) found that DEIRGs were enriched in immune pathways, such as cytokine–cytokine receptor interaction, chemokine signalling pathway, JAK-STAT signalling pathway, natural killer cell mediated cytotoxicity, and Fc epsilon RI signalling pathway.

### Potential signalling pathways were identified by GSEA

**Figure 3.**
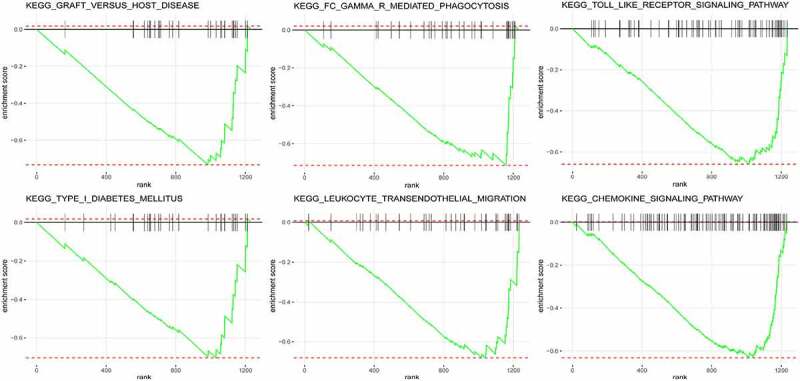
Results of the GSEA. Graft versus host disease, Fc gamma R-mediated phagocytosis, toll-like receptor signalling pathway, type 1 diabetes mellitus, leukocyte transendothelial migration, and chemokine signalling pathway were highly enriched after BS

GSEA was performed to determine the underlying mechanism by which BS may relieve inflammation. According to its normalized enrichment score, the most significant enriched signalling pathway was identified. Correlative pathways, including graft-versus-host disease, Fc gamma R-mediated phagocytosis, toll-like receptor signalling pathway, type 1 diabetes mellitus, leukocyte transendothelial migration, chemokine signalling pathway, viral myocarditis, haematopoietic cell lineage, antigen processing and presentation, cytokine–cytokine receptor interaction, and renin angiotensin system were highly enriched after BS (Figure 3 and Table 3).

**Table 3.** GSEA analysis of GSE59034

Pathway	P-value	NES	Size
KEGG_GRAFT_VERSUS_HOST_DISEASE	6.67E-04	−1.71E+00	29
KEGG_FC_GAMMA_R_MEDIATED_PHAGOCYTOSIS	5.44E-04	−1.70E+00	33
KEGG_TOLL_LIKE_RECEPTOR_SIGNALLING_PATHWAY	1.03E-04	−1.67E+00	66
KEGG_LEUKOCYTE_TRANSENDOTHELIAL_MIGRATION	1.29E-03	−1.65E+00	39
KEGG_TYPE_I_DIABETES_MELLITUS	1.89E-03	−1.64E+00	29
KEGG_CHEMOKINE_SIGNALLING_PATHWAY	1.01E-04	−1.64E+00	109
KEGG_VIRAL_MYOCARDITIS	2.21E-03	−1.64E+00	30
KEGG_HAEMATOPOIETIC_CELL_LINEAGE	1.26E-03	−1.63E+00	48
KEGG_ANTIGEN_PROCESSING_AND_PRESENTATION	3.49E-03	−1.51E+00	64
KEGG_CYTOKINE_CYTOKINE_RECEPTOR_INTERACTION	2.80E-03	−1.33E+00	229
KEGG_RENIN_ANGIOTENSIN_SYSTEM	2.91E-03	1.80E+00	5

GSEA: gene set enrichment analysis; NES: normalized enrichment score.

### PPI network analysis and identification of hub IRGs

**Figure 4.**
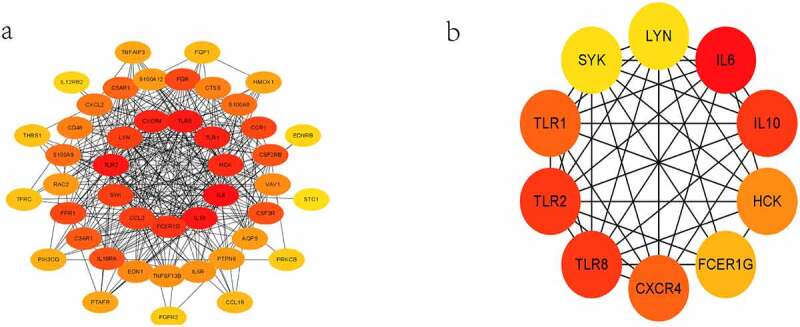
Results of the PPI and hub IRGs. PPI network of the 42 DEIRGs (a). Subnetworks of the first 10 hub IRGs from the PPI network (b)

The PPI network of DEIRGs was constructed to identify the hub IRGs (Figure 4a). In PPI network, the top 10 scoring genes were considered hub IRGs (Figure 4b and **Supplementary Table S1**). Hub IRGs were IL6, TLR8, TLR2, IL10, TLR1, CXCR4, HCK, FCER1G, LYN and SYK.

### Performance of the IRGs signature

**Figure 5.**
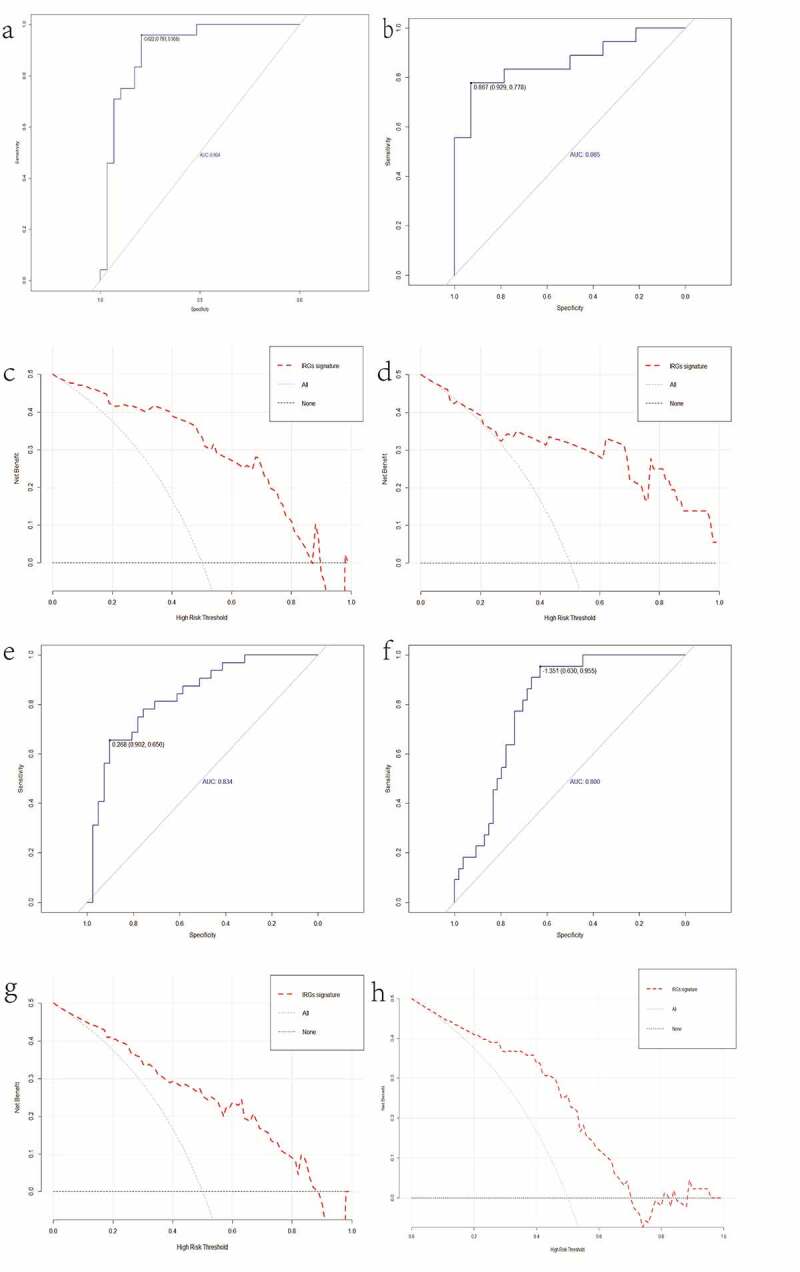
ROC curves of IRGs signature in GSE58979 (a), GSE48452 (b). Decision curve analysis (DCA) for IRGs signature in the GSE58979 (c), GSE48452 (d). ROC curves of IRGs signature in GSE48452 (e), GSE83452 (f). Decision curve analysis (DCA) for IRGs signature in the GSE48452 (g), GSE83452 (h)

The AUC of the IRGs signature was 0.904 (GSE58979) and 0.865 (GSE48452), respectively (Figure 5a, b). osmer-–Lemeshow test was not significant (0.554 and 0.710, respectively), indicating that the signature had a good fit. The IRGs signature indicated favourable prediction with a C index of 0.904 and 0.865 (training and validation cohort, respectively). DCA had shown that IRGs signature could obtain a high net benefit in predicting histological severity of NAFLD within the most reasonable threshold probability ranges (Figure 5c, d).

### Development of a NAFLD diagnostic signature based on the IRGs

To investigate the potential application of the 10 IRGs for diagnosing NAFLD, the AUC was applied to evaluate signature’ diagnostic effectiveness. The AUC and C index of GSE48452 were 0.834 (Figure 5e), and Hosmer–Lemeshow test was 0.979. The IRGs signature was established using eight hub IRGs (IL10, CXCR4, HCK, TLR2, TLR1, LYN, SYK, TLR8) and was used to determine the presence of NAFLD in the GSE83452 dataset. The AUC of IRGs signature was 0.800 (Figure 5f). Hosmer–Lemeshow test and C index were 0.353 and 0.800, respectively. DCA had shown that IRGs signature could obtain a high net benefit in diagnosing NAFLD within the most reasonable threshold probability ranges (Figure 5g, h).

### Immune cell infiltration in preoperative and postoperative BS

**Figure 6.**
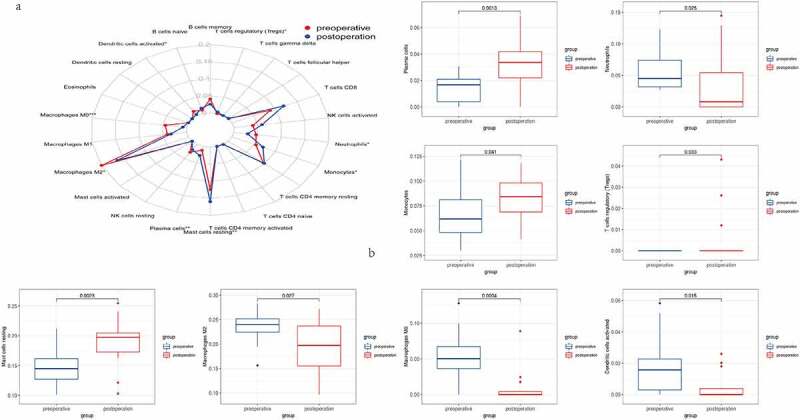
Results of immune cell infiltration. (a) The relative abundance of 22 different immune cells before and after BS. (b) Comparison of T cells regulatory (Tregs), neutrophils, macrophages M2, macrophages M0, dendritic cells activated, monocytes, mast cell resting and plasma cells before and after BS

We measured the relative abundance of 22 different immune cells in each patient before and after BS using CIBERSORT. Comparison of immune cell composition in adipose tissue before and after BS was shown in Figure 6a. The results showed that neutrophils, macrophages M2, macrophages M0 and dendritic cells activated decreased significantly after BS. However, T cells regulatory (Tregs), monocytes, mast cells resting and plasma cells infiltrated more in the adipose tissue after BS (Figure 6b).

## Discussion

Obesity can cause chronic low-grade inflammation of adipose tissue, which is manifested in various systems [26]. Obesity causes changes in adipokine production and other tissue inflammation through the proliferation and hypertrophy of adipocytes, as well as an increase in free fatty acids and extensive tissue remodelling [27]. Notably, inflammation of adipose tissue may be a prerequisite for NASH development [20]. BS is an effective treatment for obesity, reducing inflammation and NAFLD, and improving long-term survival [13,28]. However, the molecular mechanism and clinical significance of BS in improving NAFLD remain to be explored to a certain extent. Therefore, it is of profound significance to search for specific diagnostic markers and analyse the pattern of immune cell infiltration after BS to ameliorate the prognosis of NAFLD patients.

In this study, we identified 42 DEIRGs from two GEO datasets (GSE59034 and GSE29409), revealing the potential immune pathways that BS reduces adipose tissue inflammation. We developed and validated a new signature based on 10 IRGPs in multiple independent cohorts. The signature had a reliable predictive value and accuracy for predicting the histological severity of NAFLD. The results showed that BS can cause the changes of IRGs and a variety of signalling pathways in adipose tissue, and such changes may be involved in the regulation of the development of NAFLD.

Functional enrichment analysis to clarify the biological function of DEIRGs. DEIRGs were primarily enriched in chemokine signalling pathway, G-protein coupled receptor signalling pathway, JAK-STAT signalling pathway, natural killer cell mediated cytotoxicity, and Fc epsilon RI signalling pathway, etc. Confirming with previous evidence, obesity caused a low degree of chronic inflammation characterized by infiltration of B-cells, neutrophils, natural killer T cells, Th1 CD4 + T cells, macrophages, and CD8 + T cells into adipose tissue [29]. In addition to the above pathways, GSEA indicated that BS-associated IRGs were mainly enriched in Toll-like receptor signalling pathway, leukocyte transendothelial migration, antigen processing and presentation, and renin angiotensin system. These pathways have been shown to be important mechanisms of adipose tissue inflammation and to be involved in the pathogenesis of NAFLD [30–33]. More and more evidence showed that chemokine signalling pathway played an important role in the pathogenesis of NAFLD and was an important factor linking obesity and NAFLD [33]. JAK-STAT signalling pathway was involved in the regulation of inflammation associated with metabolic abnormalities in adipose tissue [34]. Mice with deficiency of Jak3 and Stat6 can display obesity and liver steatosis [35,36]. Other pathways such as Toll-like receptor, G-protein coupled receptor and natural killer cell mediated cytotoxicity were important trigger factors to promote chronic inflammation of adipose tissue and related metabolic disorders, and inhibition of these pathways played a protective role in NAFLD [30–32].

By constructing PPI network and Cytohubba analysis in Cytoscape, 10 hub IRGs were identified as IL6, TLR8, TLR2, IL10, TLR1, CXCR4, HCK, FCER1G, LYN and SYK. These genes were all downregulated after BS. The results were similar to previous studies [13,37,38]. IL-6 from adipose tissue, which travels through the portal vein to the liver, promotes NAFLD development [39]. IL10 is an anti-inflammatory cytokine released by monocytes/macrophages in adipose tissue. With the decrease of IL6 after BS, IL10 as the antagonistic anti-inflammatory cytokine of IL6 also decreased [40]. Abnormal activation of Toll-like receptors (TLRs) had been found to cause obesity-related systemic inflammation and associated comorbidities in obese rats [41]. Inhibition of TLRs could reduce the activation of MAPKs and NF-κB and ameliorate obesity-induced NAFLD [42]. CXCR4 was a G protein-coupled chemokine receptor and plays a key role in improving NAFLD after BS [43]. By increasing the affinity of CXCR4, CD4 + T-cells deposition were increased in the livers of NAFLD patients [44]. Previous study had shown that FCER1G played a key role in the occurrence and progression of NAFLD [38]. Inhibition of SYK could ameliorate the inflammation and steatosis of NAFLD by reducing the activation of macrophages and the production of CCl2, IL-6 and TNF-α [37]. Notably, FCER1G, LYN and SYK downregulation were able to reduce obesity induced by high-fat diet and the growth of lipid droplets in adipocytes [45,46]. This may be the underlying mechanism by which BS can ameliorate NAFLD.

NAFLD is one of the leading causes of liver transplantation and promotes the development of hepatocarcinoma, and BS appears to be the most durable and effective treatment for NAFLD [7,47]. BS can manipulate adipocyte-derived adipocytokines (adiponectin, leptin, IL-6, etc.) to regulate NAFLD [43]. For example, BS could inhibit the activation of Kupffer cells and hepatic stellate cells (HSC) and reduces the expression of inflammatory genes by increasing adiponectin levels [48]. In addition, Johannie DP et al. found that adipose tissue may directly promote the progression of NAFLD through inflammatory cytokines and immune cells, and they built a model using five genes to accurately predict the histological severity of NAFLD [20]. Fu C et al. recently analysed the liver tissue after BS by bioinformatic method, and they found the potential key genes and pathways for Roux-en-Y gastric bypass to ameliorate NASH [43]. However, the liver tissue samples collected include hepatocytes and mesenchymal cells, which will affect the cellular composition of the liver tissue. It has been concluded from previous studies that IRGs play an important role in obese adipose tissue and NAFLD, and inflammation in adipose tissue may be a prerequisite for NASH development. Therefore, we selected adipose tissue after BS as the training cohort to construct the signature, and liver tissue from another gene profile was selected as the validation cohort for verification. This signature had good performance in diagnosing NAFLD and its histological severity. DCA has shown that when determining NAFLD and its histological severity within the most reasonable threshold probability range, IRGs signature can provide a relatively high overall net benefit. At the same time, it also suggests that BS may ameliorate NAFLD by reducing the degree of inflammation in adipose tissue.

Increasing evidence supports the fact that immune cell infiltration plays a significant role in the initiation and progression of adipose tissue inflammation in obesity [10,49]. To further investigate the infiltration of immune cells in adipose tissue after BS, CIBERSORT algorithm was used to calculate the changes in the composition of immune cells. Neutrophils can recruit macrophages and dendritic cells to adipose tissue and play a major role in the initiation of inflammation [10]. Hypertrophy of adipocyte can secrete MCP-1/CCL2, promote the macrophages collect and produce proinflammatory cytokines TNFα, IL-6 and IL-10, resulting in NAFLD [43]. Dendritic cells in adipose tissue induce Th17 to secrete cytokines IL-6, IL-10 and IL-23, leading to more inflammation [50]. Tregs, a type of anti-inflammatory immune cells, were found that the reduction of Tregs in adipose tissue may increase the severity of NAFLD [51,52]. The above immune cell infiltration, IRGs and related pathways indicate that immune process is an important pathway for BS to treat obesity and NAFLD.

However, there were several notable limitations to this study. First, because we used data accessed from public databases, we were unable to obtain sufficient demographic and clinical information. Gender, body weight, BMI, surgical methods and other factors may influence the immune regulation mechanism of BS. Second, the follow-up time of the two datasets in this study was different. GSE59034 and GSE29409 were followed up for 2 and 1 years, respectively. In this study, some biological information may have been overlooked due to a lack of detailed consideration of follow-up time. Finally, although BS can ameliorate NAFLD, not all patients can be completely cured. The IRGs signature of this study was not used to predict the prognosis of NAFLD after BS. This limited the clinical application of the signature in this study.

In conclusion, this study analysed the potential immune mechanism of adipose tissue after BS and revealed the change of immune cells infiltration in adipose tissue after BS. In addition, this study constructed a novel IRGs signature that could accurately distinguish the severity of NAFLD. This study suggests that adipose tissue may play an important role in ameliorating NAFLD after BS.

## Supplementary Material

Supplemental Material

## Data Availability

The data that support the findings of this study are available in GEO database (http://www.ncbi.nlm.nih.gov/geo), reference number [GSE59034, GSE29409, GSE83452, GSE58979 and GSE48452].

## References

[1] NCD-RisC. Worldwide trends in body-mass index, underweight, overweight, and obesity from 1975 to 2016: a pooled analysis of 2416 population-based measurement studies in 128·9 million children, adolescents, and adults. Lancet. 2017;390(10113):2627–2642. London, England. 29029897 10.1016/S0140-6736(17)32129-3 PMC5735219

[2] Huh YJ, Seo JY, Nam J, et al. Bariatric/metabolic surgery induces noticeable changes of microbiota and their secreting extracellular vesicle composition in the gut. Obes Surg. 2019;29(8):2470–2484. 31129882 10.1007/s11695-019-03852-1

[3] Ng M, Fleming T, Robinson M, et al. Global, regional, and national prevalence of overweight and obesity in children and adults during 1980-2013: a systematic analysis for the global burden of disease study 2013. Lancet. 2014;384(9945):766–781. London, England. 24880830 10.1016/S0140-6736(14)60460-8 PMC4624264

[4] Haas JT, Vonghia L, Mogilenko DA, et al. Transcriptional Network Analysis Implicates Altered Hepatic immune function in NASH development and resolution. Nat Metab. 2019;1(6):604–614. 31701087 10.1038/s42255-019-0076-1 PMC6837876

[5] Brunt EM. Pathology of nonalcoholic fatty liver disease. Nat Clin Pract Gastroenterol Hepatol. 2010;7(4):195–203. 10.1038/nrgastro.2010.21 20195271

[6] Goossens N, Hoshida Y, Song WM, et al. Nonalcoholic steatohepatitis is associated with increased mortality in obese patients undergoing bariatric surgery. Clin Gastroenterol Hepatol. 2016;14(11):1619–1628. 26492845 10.1016/j.cgh.2015.10.010 PMC4838546

[7] Younossi Z, Anstee QM, Marietti M, et al. Global burden of NAFLD and NASH: trends, predictions, risk factors and prevention. Nat Clin Pract Gastroenterol Hepatol. 2018;15(1):11–20. 10.1038/nrgastro.2017.109 28930295

[8] Mathis D. Immunological goings-on in visceral adipose tissue. Cell Metab. 2013;17(6):851–859. 23747244 10.1016/j.cmet.2013.05.008 PMC4264591

[9] Stanton MC, Chen SC, Jackson JV, et al. Inflammatory signals shift from adipose to liver during high fat feeding and influence the development of steatohepatitis in mice. J Inflam. 2011;8(1):8. London, England. 10.1186/1476-9255-8-8 PMC3070617 21410952

[10] Poitou C, Perret C, Mathieu F, et al. Bariatric surgery induces disruption in inflammatory signaling pathways mediated by immune cells in adipose tissue: a RNA-Seq study. PloS One. 2015;10(5):e0125718. 25938420 10.1371/journal.pone.0125718 PMC4418598

[11] Schauer PR, Bhatt DL, Kirwan JP, et al. Bariatric surgery versus intensive medical therapy for diabetes–3-year outcomes. N Engl J Med. 2014;370(21):2002–2013. 24679060 10.1056/NEJMoa1401329 PMC5451259

[12] Chen Y, Yang J, Nie X, et al. Effects of bariatric surgery on change of brown adipocyte tissue and energy metabolism in obese mice. Obes Surg. 2018;28(3):820–830. 28853013 10.1007/s11695-017-2899-8

[13] Liu Y, Jin J, Chen Y, et al. Integrative analyses of biomarkers and pathways for adipose tissue after bariatric surgery. Adipocyte. 2020;9(1):384–400. 32684073 10.1080/21623945.2020.1795434 PMC7469525

[14] Laursen TL, Hagemann CA, Wei C, et al. Bariatric surgery in patients with non-alcoholic fatty liver disease - from pathophysiology to clinical effects. World J Hepatol. 2019;11(2):138–149. 30820265 10.4254/wjh.v11.i2.138 PMC6393715

[15] Guo D, Wang M, Shen Z, et al. A new immune signature for survival prediction and immune checkpoint molecules in lung adenocarcinoma. J Transl Med. 2020;18(1):123. 32143735 10.1186/s12967-020-02286-z PMC7060601

[16] Zhu R, Tao H, Lin W, et al. Identification of an immune-related gene signature based on immunogenomic landscape analysis to predict the prognosis of adult acute myeloid leukemia patients. Front Oncol. 2020;10:574939. 33330048 10.3389/fonc.2020.574939 PMC7714942

[17] Petrus P, Mejhert N, Corrales P, et al. Transforming growth factor-β3 regulates adipocyte number in subcutaneous white adipose tissue. Cell Rep. 2018;25(3):551–60.e5. 30332637 10.1016/j.celrep.2018.09.069

[18] Hoggard N, Cruickshank M, Moar KM, et al. Using gene expression to predict differences in the secretome of human omental vs. subcutaneous adipose tissue. Obesity. 2012;20(6): 1158–1167. Silver Spring, Md. 22286531 10.1038/oby.2012.14

[19] Lefebvre P, Lalloyer F, Baugé E, et al. Interspecies NASH disease activity whole-genome profiling identifies a fibrogenic role of PPARα-regulated dermatopontin. JCI Insight. 2017;2(13):13. 10.1172/jci.insight.92264 PMC5499370 28679947

[20] Du Plessis J, Van Pelt J, Korf H, et al. Association of adipose tissue inflammation with histologic severity of nonalcoholic fatty liver disease. Gastroenterology. 2015;149(3):635–48.e14. 26028579 10.1053/j.gastro.2015.05.044

[21] Ahrens M, Ammerpohl O, von Schönfels W, et al. DNA methylation analysis in nonalcoholic fatty liver disease suggests distinct disease-specific and remodeling signatures after bariatric surgery. Cell Metab. 2013;18(2):296–302. 23931760 10.1016/j.cmet.2013.07.004

[22] Jia X, Zhai T. Integrated analysis of multiple microarray studies to identify novel gene signatures in non-alcoholic fatty liver disease. Front Endocrinol (Lausanne). 2019;10:599. 31551930 10.3389/fendo.2019.00599 PMC6736562

[23] Wilson PC, Wu H, Kirita Y, et al. The single-cell transcriptomic landscape of early human diabetic nephropathy. Proc Natl Acad Sci U S A. 2019;116(39):19619–19625. 31506348 10.1073/pnas.1908706116 PMC6765272

[24] Chin CH, Chen SH, Wu HH, et al. cytoHubba: identifying hub objects and sub-networks from complex interactome. BMC Syst Biol. 2014;8(Suppl 4):S11. 25521941 10.1186/1752-0509-8-S4-S11 PMC4290687

[25] Newman AM, Steen CB, Liu CL, et al. Determining cell type abundance and expression from bulk tissues with digital cytometry. Nat Biotechnol. 2019;37(7):773–782. 31061481 10.1038/s41587-019-0114-2 PMC6610714

[26] Rogero MM, Calder PC. Obesity, inflammation, toll-like receptor 4 and fatty acids. Nutrients. 2018;10(4):4. 10.3390/nu10040432 PMC5946217 29601492

[27] Kolb R, Sutterwala FS, Zhang W. Obesity and cancer: inflammation bridges the two. Curr Opin Pharmacol. 2016;29:77–89. 27429211 10.1016/j.coph.2016.07.005 PMC4992602

[28] Zhang C, Zhang J, Liu Z, et al. More than an anti-diabetic bariatric surgery, metabolic surgery alleviates systemic and local inflammation in obesity. Obes Surg. 2018;28(11):3658–3668. 30187424 10.1007/s11695-018-3400-z

[29] Lee BC, Lee J. Cellular and molecular players in adipose tissue inflammation in the development of obesity-induced insulin resistance. Biochim Biophys Acta. 2014;1842(3):446–462. 23707515 10.1016/j.bbadis.2013.05.017 PMC3800253

[30] Saad ZA, Khodeer DM, Zaitone SA, et al. Exenatide ameliorates experimental non-alcoholic fatty liver in rats via suppression of toll-like receptor 4/NFκB signaling: comparison to metformin. Life Sci. 2020;253:117725. 32348835 10.1016/j.lfs.2020.117725

[31] Wang J, Ma J, Nie H, et al. Hepatic regulator of G protein signaling 5 ameliorates nonalcoholic fatty liver disease by suppressing transforming growth factor beta-activated Kinase 1-c-Jun-N-Terminal Kinase/p38 signaling. Hepatology. 2021;73(1):104–125. Baltimore, Md. 10.1002/hep.31242 32191345

[32] Cuff AO, Sillito F, Dertschnig S, et al. The obese liver environment mediates conversion of NK cells to a less cytotoxic ILC1-Like phenotype. Front Immunol. 2019;10:2180. 31572388 10.3389/fimmu.2019.02180 PMC6749082

[33] Roh YS, Seki E. Chemokines and chemokine receptors in the development of NAFLD. Adv Exp Med Biol. 2018;1061:45–53. 29956205 10.1007/978-981-10-8684-7_4

[34] Dodington DW, Desai HR, Woo M. JAK/STAT - emerging players in metabolism. Trends Endocrinol Metab. 2018;29(1):55–65. 29191719 10.1016/j.tem.2017.11.001

[35] Mishra J, Verma RK, Alpini G, et al. Role of janus kinase 3 in predisposition to obesity-associated metabolic syndrome. J Biol Chem. 2015;290(49):29301–29312. 26451047 10.1074/jbc.M115.670331 PMC4705936

[36] INVALID CITATION.

[37] Kurniawan DW, Jajoriya AK, Dhawan G, et al. Therapeutic inhibition of spleen tyrosine kinase in inflammatory macrophages using PLGA nanoparticles for the treatment of non-alcoholic steatohepatitis. J Control Release. 2018;288:227–238. 30219279 10.1016/j.jconrel.2018.09.004

[38] Dai W, Sun Y, Jiang Z, et al. Key genes associated with non-alcoholic fatty liver disease and acute myocardial infarction. Med Sci Monit. 2020;26:e922492. 32594092 10.12659/MSM.922492 PMC7341693

[39] Adam S, Liu Y, Siahmansur T, et al. Bariatric surgery as a model to explore the basis and consequences of the reaven hypothesis: small, dense low-density lipoprotein and interleukin-6. Diab Vasc Dis Res. 2019;16(2):144–152. 31014098 10.1177/1479164119826479

[40] Modolin MLA, Cintra W Jr., Rocha RI, et al. Analysis of inflammatory and metabolic biomarkers in patients submitted to abdominoplasty after bariatric surgery. Acta Cir Bras. 2019;34(5):e201900506. 31166465 10.1590/s0102-865020190050000006 PMC6583934

[41] Sala P, Torrinhas R, Fonseca DC, et al. Intestinal expression of toll-like receptor gene changes early after gastric bypass surgery and association with type 2 diabetes remission. Nutrition. Burbank, Los Angeles County, Calif 2020;79-80: 110885. 32707229 10.1016/j.nut.2020.110885

[42] Wu L, Sun J, Liu L, et al. Anti-toll-like receptor 2 antibody ameliorates hepatic injury, inflammation, fibrosis and steatosis in obesity-related metabolic disorder rats via regulating MAPK and NF-κB pathways. Int Immunopharmacol. 2020;82:106368. 32151955 10.1016/j.intimp.2020.106368

[43] Chen F, Zhou Y, Wu Z, et al. Integrated analysis of key genes and pathways involved in nonalcoholic steatohepatitis improvement after roux-en-Y gastric bypass surgery. Front Endocrinol (Lausanne). 2020;11:611213. 33603714 10.3389/fendo.2020.611213 PMC7884850

[44] Boujedidi H, Robert O, Bignon A, et al. CXCR4 dysfunction in non-alcoholic steatohepatitis in mice and patients. Clin Sci. 2015;128(4):257–267. London, England: 1979. 10.1042/CS20130833 25074471

[45] van Beek L, Vroegrijk IO, Katiraei S, et al. FcRγ-chain deficiency reduces the development of diet-induced obesity. Obesity (Silver Spring, Md). 2015;23(12):2435–2444. 10.1002/oby.21309 26523352

[46] Hao JW, Wang J, Guo H, et al. CD36 facilitates fatty acid uptake by dynamic palmitoylation-regulated endocytosis. Nat Commun. 2020;11(1):4765. 32958780 10.1038/s41467-020-18565-8 PMC7505845

[47] Lassailly G, Caiazzo R, Buob D, et al. Bariatric surgery reduces features of nonalcoholic steatohepatitis in morbidly obese patients. Gastroenterology. 2015;149(2):379–388. 25917783 10.1053/j.gastro.2015.04.014

[48] Braunersreuther V, Viviani GL, Mach F, et al. Role of cytokines and chemokines in non-alcoholic fatty liver disease. World J Gastroenterol. 2012;18(8):727–735. 22371632 10.3748/wjg.v18.i8.727 PMC3286135

[49] Maurizi G, Della Guardia L, Maurizi A, et al. Adipocytes properties and crosstalk with immune system in obesity-related inflammation. J Cell Physiol. 2018;233(1):88–97. 28181253 10.1002/jcp.25855

[50] Russo L, Lumeng CN. Properties and functions of adipose tissue macrophages in obesity. Immunology. 2018;155(4):407–417. 30229891 10.1111/imm.13002 PMC6230999

[51] Jalilvand A, Blaszczak A, Bradley D, et al. Low visceral adipose tissue regulatory T cells are associated with higher comorbidity severity in patients undergoing bariatric surgery. Surg Endosc. 2021;35(6):3131-8. 10.1007/s00464-020-07751-w 32572626

[52] Vonghia L, Ruyssers N, Schrijvers D, et al. CD4+ROR γ t++ and tregs in a mouse model of diet-induced nonalcoholic steatohepatitis. Mediators Inflamm. 2015;2015:239623. 10.1155/2015/239623 PMC4503578 26229237

