# Supplementary material for: A novel immune-related genes signature after bariatric surgery is histologically associated with non-alcoholic fatty liver disease
Source: Adipocyte. 2021 Sep 10;10(1):424–34. doi: 10.1080/21623945.2021.1970341 (PMC8437528; doi:10.1080/21623945.2021.1970341)
Supplement: Supplemental Material [file KADI_A_1970341_SM5155.zip › S table 1.doc]

| Supplementary Table 1 Hub IRGs in PPI network ranked by Degree method | | |
| --- | --- | --- |
| Rank | Name | Score |
| 1 | IL6 | 31 |
| 2 | TLR8 | 28 |
| 2 | TLR2 | 28 |
| 2 | IL10 | 28 |
| 5 | TLR1 | 25 |
| 5 | CXCR4 | 25 |
| 7 | HCK | 24 |
| 8 | FCER1G | 21 |
| 9 | LYN | 20 |
| 10 | SYK | 20 |
